# Saturation in qualitative research: An evolutionary concept analysis

**DOI:** 10.1016/j.ijnsa.2024.100174

**Published:** 2024-01-05

**Authors:** Sara Rahimi, Marzieh khatooni

**Affiliations:** a Social Determinants of Health Research Center, Research Institute for Prevention of Non-Communicable Diseases, Qazvin University of Medical Sciences, Qazvin, Iran; b Faculty of Nursing and Midwifery, Qazvin University of Medical Sciences, Qazvin, Iran

**Keywords:** Saturation, Data collection, Qualitative research, Sample size, Concept analysis

## Abstract

**Background and Aim:**

Qualitative research plays an important role in improving nursing knowledge. Understanding the concept of saturation is essential to conducting rigorous qualitative research that contributes to evidence-based practice. The purpose of this study is to clarify the concept of saturation in qualitative research.

**Method:**

Evolutionary concept analysis was performed. A literature search was conducted using a variety of online databases for the years 2005- 2023. In total, 33 articles and books were analyzed using thematic analysis to identify the attributes, antecedents and consequences of saturation. The validity of the data was obtained by examining the analysis process by two independent researchers.

**Results:**

Saturation in qualitative research is a context-dependent, subjective process that requires detailed systematic analysis. Saturation is used in four ways in qualitative research: theoretical saturation, data saturation, code or thematic saturation, and meaning saturation. The antecedents of saturation were classified into two categories: study related factors and researcher related factors. The consequences of saturation were identified as: ensuring credibility and quality in qualitative research and time, energy and budget saving.

**Conclusion:**

This concept analysis serves to enhance the understanding of the concept of saturation, thereby offering valuable resources for qualitative researchers. By gaining a profound comprehension of saturation and its various types, researchers can ensure the validity of their studies while also optimizing time and resource allocation by avoiding redundant data collection. Future investigation warranted to elucidate how factors associated with reaching saturation impact estimations sample size.

## What is already known

• The concept of saturation is one of the important aspects of qualitative research. It is a criterion for stopping data collection and/or analysis in qualitative research.
• A deep understanding of the concept of saturation by researchers is necessary to ensure the validity and quality of qualitative research.
• The lack of clarity and debate over the meaning of saturation often causes researchers to not provide clear explanations for its definition and determination in their work.

## What this paper adds

• This is the first concept analysis performed on the concept of saturation in qualitative research.
• Elucidating the concept of saturation provides a valuable contribution to the field of qualitative research methodology.
• This study provides valuable insight for qualitative researchers by clarifying the concept of saturation, its characteristics, types, and factors influencing its achievement.

## 1 Introduction

Qualitative research has become more popular in the last decades and has been widely accepted in most disciplines, such as psychology, sociology, medicine, business, economics, and anthropology (Mwita, 2022; Thomson, 2010). One of the important aspects of qualitative research is the concept of saturation. Saturation is a criterion for discontinuing data collection and/or analysis in qualitative research (Saunders et al., 2018). The concept of saturation was first described by Glaser and Strauss (1967) in the context of grounded theory. They stated that saturation is necessary for the development of a theory that accurately reflects the reality of the phenomenon under study (Glaser & Strauss, 1967). The term used in grounded theory is theoretical saturation. Theoretical saturation is achieved when no additional themes or insights emerge from the data collection, and all conceptual categories have been explored, identified, and completed (Hennink et al., 2017).

Although the concept of saturation originates in grounded theory, it is also used in many other approaches to qualitative research. Indeed, saturation is often suggested as an essential methodological element in qualitative research. (Hennink et al., 2017; Saunders et al., 2018). Achieving saturation is a critical component of qualitative research and is often promoted as a means to ensure research rigor (Morse, 2015a). Therefore, failure to achieve saturation definitely affects the quality of the conducted research (Fusch & Ness, 2015). Saturation is an important indicator of whether a sample is sufficient for the phenomenon under study—that the data collected represents the diversity, depth, and nuances of the subjects studied—and thereby represents content validity (Francis et al., 2010). However, adopting this concept from its methodological context and applying it in other qualitative research, despite its acceptance, is still associated with problems. When the concept of saturation is taken from the context of grounded theory, without adequate guidance on its application in a broader context, it is not clear what it means and how it can be achieved (Kerr et al., 2010).

Lack of clarity and the debates over the meaning of saturation often cause qualitative researchers not to provide clear explanations for its definition and determination in their work (Alam, 2021; Bowen, 2008; Morse, 2015b). In some cases, they even limit its definition to merely a footnote within the articles (Alam, 2021; Bowen, 2008). The results of a systematic review of 220 qualitative studies showed that even though 80% of studies had used the word saturation to justify their sample size, they presented superficial reports of the explanation of saturation and how to achieve it (Carlsen & Glenton, 2011). Francis et al. (2010) reviewed qualitative articles published in a journal over 16 months. They found that despite the claim of reaching saturation in 15 articles of the 18 articles that mentioned data saturation, the researchers did not provide a clear explanation about how saturation was defined, achieved, or justified in their studies (Francis et al., 2010).

Researchers’ ambiguity regarding saturation application, and the lack of explicit guidelines for its determination, creates a potential problem for evaluating the quality of research (Bowen, 2008; Rowlands et al., 2016). Saturation has different meanings depending on qualitative research approaches (O'reilly & Parker, 2013). Therefore, it is inappropriate to use it as a general indicator for sample adequacy, regardless of how it is evaluated and what it means for different types of studies and different types of data (Hennink et al., 2017). A deep understanding of the concept of saturation by the researchers is necessary to ensure the credibility and quality of qualitative research. Hence, it is essential that researchers in their studies provide a clear definition of saturation, evidence of saturation in data presentation, and the process of achieving it during data analysis (Caelli et al., 2003). This is while novice researchers are facing many problems in defining, identifying, and determining saturation because there are almost no clear strategies for defining saturation in the literature on qualitative methods (Alam, 2021).

One of the methods used in qualitative research to clarify a concept is concept analysis. Concept analysis explores the description, application, and measurement of a concept, facilitating its optimal use and evaluation by providing a detailed description of the concept and its conditions. Through concept analysis, researchers gain a deeper understanding of the concept and its practical applications, contributing to the refinement and advancement of knowledge within the field of study (Rogers, 2000; Tofthagen & Fagerstrøm, 2010).

In this study, we intend to analyze the concept of saturation, to identify its characteristics, types, and factors influencing its attainment. This information can provide valuable insights into understanding saturation in qualitative research for qualitative researchers, postgraduate students, and instructors who teach or supervise qualitative research projects. In addition, by clarifying the concept of saturation, this study can contribute to the growing body of knowledge on qualitative research methodology.

## 2 Methods

This study is a concept analysis. Rogers' evolutionary method was chosen to analyze the concept of saturation due to its emphasis on inductive inquiry and detailed analysis compared to other methods. The strength of this method is that it is systematic and focuses on clear steps in the analysis process (Tofthagen & Fagerstrøm, 2010). The six steps of this method are shown in Table 1 (Table 1).

**Table 1:** Steps in the Rodgers’ Evolutionary Method for Concept Analysis (Rogers, 2000).

Rogers states that these steps are not necessarily presented linearly in a study, and one step may be integrated into another step or steps (Rogers, 2000). In this study, the step of identifying the implications of the concept has been integrated into the steps of identifying surrogate and related expressions (stage 1) and analyzing data regarding attributes, antecedents, and consequences (stages 2 and 3). Because in these steps the implications of saturation have been discussed.

### 2.1 Identification of the concept of interest

Tofthagen & Fagerstrøm (2010) state that in Rogers' method, "significance" is a central aspect in selecting a concept for analysis. They express the importance of analyzing a concept to serve a purposeful human goal and helping to solve a problem (Tofthagen & Fagerstrøm, 2010). Hence, considering the necessity of clarification of saturation in qualitative research, this concept was chosen for analysis. Saturation is used in various methods of qualitative research; hence Rogers' evolutionary approach was an appropriate approach for analyzing the concept of saturation due to its context-based nature.

### 2.2 Identification and selection of an appropriate setting and sample for data collection

**Fig. 1:**
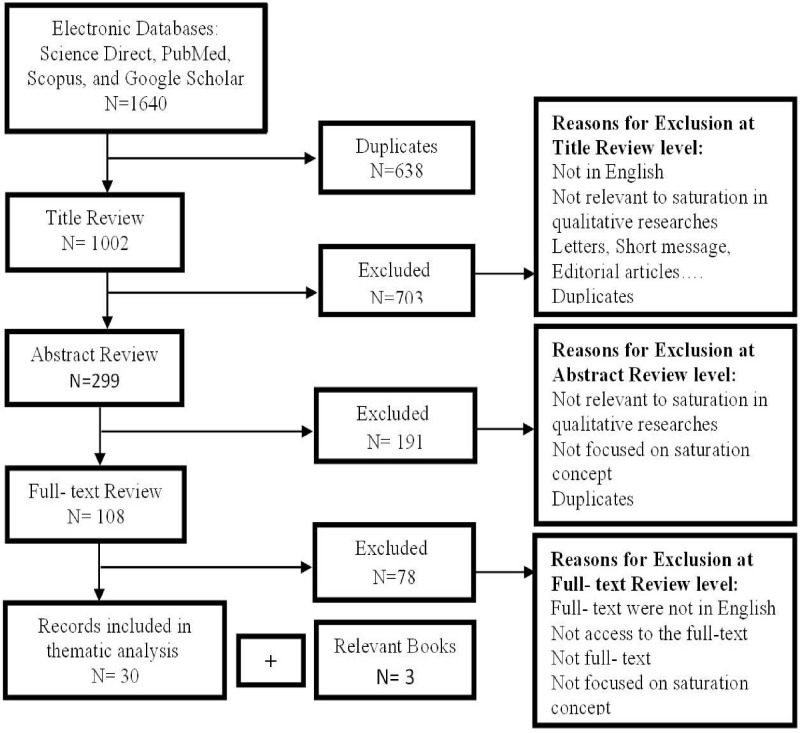
Summary of the literature search and the number of articles.

The context in which a concept is used affects its meaning and application. To analyze a concept effectively, Rogers (2000) recommends deffining a context (Rogers, 2000). In this study, the concept of saturation was analyzed in the context of "qualitative research". Given the growth of conducting qualitative studies since 2005, the period from 2005 to March 2023 was selected for sampling. For this purpose, large databases such as Science Direct, Pub Med, Scopus, and Google Scholar were searched to find related articles or book chapters published during this period. The search was done in the title, abstract, and key words of articles. The terms: "Saturation", "Theoretical saturation", "Data saturation", "Grounded Theory", "sample size", "data collection", and "qualitative research" alone or in combination using Boolean (AND/OR) were searched. Both authors independently screened all article titles, abstracts, and, if needed, full texts to determine eligibility for analysis. Disagreements were discussed and resolved by consensus. Articles related to the desired concept from all disciplines were examined. Articles that were in English and whose title, abstract, or main questions related to saturation, were included in the study. At the same time, duplicated and letters, short message, editorial articles, and those containing saturation terms outside of the qualitative research context, were excluded. Finally, 30 articles and 3 relevant books were included in the concept analysis. The summary of the literature search and the number of articles are shown in Fig. 1. (See Fig. 1).

### 2.3 Data Analysis

In Rogers' method, thematic analysis is used. For this purpose, all the selected articles were first numbered in order of publication date. Then they were carefully examined in depth, using questions such as "What are the characteristics of this concept?" "What happens when the desired concept occurs?" "What are the preconditions for the concept to occur? And "What consequences does this concept bring?" Then, the obtained data related to the main categories, which included attributes, antecedents, and consequences of saturation, were recorded on separate sheets of paper. Finally, the data written on each sheet of paper was categorized into subcategories and the developed subcategories were labeled. Both authors agreed on the categorization.

## 3 Results and Discussion

### 3.1 Identification of surrogate and related terms

Surrogate terms are words that express the ideas of a concept through words other than the concept that the researcher has chosen in his/her study. Related terms are words that are common with the concept in some features but do not have the same characteristics (Rogers, 2000). Saturation is a widely used concept in all types of qualitative research, which has different meanings depending on the type of study, research question, theoretical framework and assumptions of the study (Faulkner & Trotter, 2017; Saunders et al., 2018). The literature review revealed that researchers used concepts such as theoretical saturation (Aldiabat & Le Navenec, 2018; Bowen, 2008; Charmaz, 2006; Faulkner & Trotter, 2017; Glaser & Strauss, 1967; Hennink & Kaiser, 2022; Hennink et al., 2017; Kerr et al., 2010; Saunders et al., 2018; Thomson, 2010) data saturation (Kerr et al., 2010; Saunders et al., 2018), code saturation (Hennink et al., 2017; Saunders et al., 2018), thematic saturation (Bowen, 2008; Faulkner & Trotter, 2017; Guest et al., 2006; Guest et al., 2020; Hennink & Kaiser, 2022; Hennink et al., 2017; Kerr et al., 2010; Low, 2019; Saunders et al., 2018) and, meaning saturation as a surrogate term, were used in qualitative studies. Also, redundancy of information and/or data (Bowen, 2008; Braun & Clarke, 2021; Faulkner & Trotter, 2017; Marshall et al., 2013), overload of information and/or data, and pseudo-saturation (Aldiabat & Le Navenec, 2018) were terms related to saturation.

### 3.2 Theoretical saturation

Glaser and Strauss (1967) defined theoretical saturation in the field of grounded theory as the point at which no additional data is found that would allow the researcher to further develop the properties of the phenomenon (Glaser & Strauss, 1967). Charmaz later expanded on this concept and noted that it is not merely about collecting more data to generate new themes, but rather a process of actively searching for and testing supporting evidence for the emerging theory. The researcher continues data collection until the emerging theory becomes strong and accounts for all the evidence (Bryant & Charmaz, 2007). Therefore, theoretical saturation is a point where further data collection about a theoretical structure does not reveal any new features or provide additional theoretical insight into the emerging theory. An important aspect of theoretical saturation is that in an iterative process, the researcher simultaneously engages in sampling, data collection, and data analysis (Sandelowski, 1995). This characteristic requires the use of theoretical sampling. Theoretical sampling involves guiding and selecting subsequent participants based on the concepts identified from current data analysis, and it continues until theoretical saturation is reached. Theoretical sampling and theoretical saturation are inseparable, and they persist until all the structures of a phenomenon, such as themes, concepts, categories, and relationships, are fully identified and the emerging theory is completed (theoretical saturation) (Guest et al., 2006; Hennink et al., 2017).

### 3.3 Data saturation

The definition of saturation by Glaser and Strauss specifically focused on theory construction and theoretical models, while many qualitative data analyses do not use grounded theory methodology. Over time, qualitative researchers have adopted the term "data saturation". Data saturation reflects a broader application and describes a point in data collection and analysis where new data or information does not contribute significantly to addressing the research question, or when existing data is replicated (Guest et al., 2006; Saunders et al., 2018). Although the concept of data saturation is often used to refer generally to saturation in qualitative research, since its focus is on the emergence of new information from the data, judgment about it relates to the data collection stage. Therefore, researchers may acknowledge the attainment of data saturation when no new data is obtained in this stage, although such a judgment is often misleading. This is because during the data analysis process and throughout qualitative work, codes, code properties, and the relationships between them undergo changes such as code merging, expansion, and elimination, necessitating further data collection. Thus, relying solely on data saturation as the sole criterion for judging saturation is accompanied by inherent limitations (Saunders et al., 2018; Yang et al., 2022).

### 3.4 Code or thematic saturation

Code or thematic saturation refers to a stage in the data analysis process where repetitive codes or themes are identified, and no new information or relationships between them emerge (Bowen, 2008; Guest et al., 2020; Hennink et al., 2017; Low, 2019; Saunders et al., 2018; Yang et al., 2022). Code and thematic saturation is sometimes called category saturation (Yang et al., 2022). This type of saturation improves reliability compared to data saturation, which is often limited to the data collection stage and focuses solely on data repeatability. This is because coding and theme development require a certain level of analysis, such that coding enters the data analysis stage and the emergence of themes is the result of achieving a certain level of coding (Saunders et al., 2018; Yang et al., 2022). However, using it as a basis for judging data saturation is still susceptible to false saturation due to the lack of discovery of relationships between codes and obtained themes, and still requires more data for the researcher to fully understand the depth, richness, and complexity of the phenomenon (Hennink et al., 2017; Low, 2019; Saunders et al., 2018). This is particularly important when the researcher is confronted with a topic that is emerging from the data for the first time and lacks a deep understanding, as further data collection and analysis are needed to create a richer and deeper understanding of the studied phenomenon (Hennink et al., 2017; Kerr et al., 2010).

### 3.5 Meaning saturation

Meaning saturation has been introduced by Hennink et al., as a point in the process of data collection and analysis where issues are fully understood and no new information about the meaning of codes or themes and their relationships emerges. Meaning saturation is based on judgment regarding the completeness of coding and themes and the deep understanding of data (Hennink et al., 2017). This means that simply stating that codes or themes have been repeated and no new code or theme has been found is not enough (Kerr et al., 2010), and the explanatory and interpretive dimension of each code or theme must start repeating and no further explanation is found to claim that saturation has been achieved (Yang et al., 2022). Therefore, achieving meaning saturation requires an iterative and cyclical process of sampling, data collection, and analysis, continuous monitoring of the diversity, clarity, and depth of data.

Despite researchers' agreement on the general principles of saturation, including not obtaining new data, themes, and codes, as well as the ability to repeat the study (Fusch & Ness, 2015; Guest et al., 2006), different types of saturation differ in terms of focus on the research stage and the judgment of when it is reached. While data saturation is focused on the process of data collection, code or thematic saturation is focused on primary data analysis, and meaning saturation is focused on data analysis; theoretical saturation is at a higher level and emphasizes the adequacy of discovering conceptual themes and their theoretical meanings during theory building process. Different types of saturation are based on different judgments. Data, code, or thematic saturation is judged based on the repetition or frequency of data, codes, or themes. On the other hand, meaning saturation is judged based on the depth of meaning in themes, and theoretical saturation is judged based on the completeness of concept features and the coherence and relationships formed with other concepts and the overall theory (Hennink et al., 2017; Saunders et al., 2018; Yang et al., 2022). Generally, data, code, or thematic saturation focuses on the breadth of collected data, while meaning saturation and theoretical saturation focus on the depth of research data (Conlon et al., 2020; Saunders et al., 2018). However, meaning saturation focuses on the depth of meaning in themes and can be used in various qualitative research studies, while theoretical saturation focuses on the completeness, predictability, and explanatory power of emerging theories and is mainly applicable in grounded theory (Hennink et al., 2017; Yang et al., 2022). It should be noted that presenting different types of saturation does not imply that one type is always applicable in a study. Researchers have the flexibility to integrate two or more types of saturation as needed. However, familiarity with these types helps them choose relevant saturation types depending on their research methodology (Saunders et al., 2018). Table 2 summarizes the types of saturation, their main focus, and judgment stage in the research process (See Table 2).

**Table 2:** Types of saturation, their main focus, and judgment stage in the research process.

### 3.6 Terms related to saturation

#### 3.6.1 Information and/or data redundancy

Information and/or data redundancy is a concept related to saturation in qualitative research. However, its meaning differs to some extent from saturation. The concept of saturation is often defined as information redundancy, albeit in a broad and weak sense (Lincoln & Guba, 1985), while redundancy refers to encountering repetitive data or collected information, and saturation is reaching a point where no new information is obtained from this repetitive data. Information redundancy helps conceptualize saturation (Alam, 2021; Braun & Clarke, 2021; Bryman, 2006).

#### 3.6.2 Information or data overload

Information or data overload refers to a massive volume of information that a researcher may encounter during their analysis, making it difficult to identify key themes and patterns. Information overload can lead to confusion and incapability in understanding the data, and the difference between it and data saturation and information redundancy is that, unlike the latter two where the researcher has collected sufficient data and can proceed towards their analysis, in information overload, the researcher is faced with a large volume of information (which may also be insufficient) that poses challenges in starting the analysis (Bawden & Robinson, 2020; Lee Eden, 2022).

#### 3.6.3 Pseudo-saturation

Pseudo-saturation is a concept that is used to describe a situation in qualitative research where the researcher mistakenly believes that they have reached saturation, but in reality, there is still new or additional information to be obtained. There are several reasons for pseudo-saturation, such as prematurely ending data collection, not exploring different perspectives or contexts, or overlooking important sources of information (Aldiabat & Le Navenec, 2018; Guest et al., 2006; Morse, 2015a).

#### 3.6.4 Analysis of the data regarding the attributes of the concept

Attributes are features of a concept. They constitute the actual meaning of the concept in comparison to its nominal definition and provide an opportunity to identify the conceptual field. The researcher carefully examines literature in depth, using question "What are the characteristics of this concept?" (Rogers, 2000). The attributes of saturation in this study were categorized into four themes: process-oriented, context-dependent, subjective, and requiring systematic and precise analysis.

#### 3.6.5 rocess-oriented concept

Data saturation is a process-oriented concept, meaning that there is no specific point at which no new information can be obtained from additional data. Instead, it is a gradual process, and there is no specific point at which data saturation occurs. Rather, there are a range of points at which researchers may feel satisfied with the amount of data they have collected (Guest et al., 2006; Saunders et al., 2018). Dey (1999) views saturation as an accumulative and ongoing judgment by the researcher that may never be complete (Dey, 1999). This perspective aligns with Glaser and Strauss's (1967) view of theory construction, which suggests that the process of theory generation is endless, and researchers may continue to modify and develop aspects of their emerging theory even when reviewing their study for publication (Glaser & Strauss, 1967). Therefore, some studies recommend that even after reaching saturation, researchers continue to collect additional data (one or two interviews or focus groups) to validate or expand their findings (Francis et al., 2010; Morse, 2015a). These additional interviews can help validate patterns, concepts, themes, and dimensions identified in previous interviews (Thomson, 2010).

#### 3.6.6 Context-dependent concept

Data saturation is a contextually dependent concept, and achieving saturation in different research studies can vary depending on the research question, methodology, and data collection methods (Guest et al., 2006; Guest et al., 2020; Marshall et al., 2013; Saunders et al., 2018). Contextual dependence means that meaning is constructed within a specific context and cannot be separated from that context (Denzin & Lincoln, 2011). Therefore, the interpretation of data in qualitative research is influenced by the context in which the data is collected and analyzed. Researchers need to be clear in explaining how data was collected and how saturation was reached. Before using a research method, the researcher needs to understand the philosophical underpinning of their research tradition because the understanding of the ontological and epistemological beliefs used affects their orientation towards the nature of reality, what can be known and how it can be known (Aldiabat & Le Navenec, 2018; Fusch & Ness, 2015; Guest et al., 2020; Hennink et al., 2017). The concept of saturation has a close connection with epistemological and ontological assumptions that underpin qualitative research. For example, the constructivist and interpretive paradigms emphasize the importance of understanding experiences and mental meanings. In this view, saturation is a dynamic process that is subject to the researcher's interpretation of the data and may require collecting more extensive data to achieve saturation (Charmaz, 2006; Fusch & Ness, 2015). In contrast, the positivist paradigm prioritizes objectivity and generalizability and sees saturation as a point that can be reached through precise data analysis (Bowen, 2008; Creswell & Poth, 2016), so it may be possible to achieve saturation with a smaller sample size (Creswell & Poth, 2016).

#### 3.6.7 Subjective concept

Subjectivity refers to the idea that achieving saturation can be dependent on the researcher's interpretation of the data. Qualitative research is not immune to biases, assumptions, and researchers' perspectives, and different researchers may interpret the same data differently based on their perspectives and experiences (Braun & Clarke, 2021; Creswell & Poth, 2016; Guest et al., 2020; Kerr et al., 2010; Marshall et al., 2013; Mwita, 2022; Rowlands et al., 2016). Therefore, a researcher who has preconceived ideas about a specific topic may reach saturation earlier compared to a researcher who approaches the same topic with an open mind. The subjectivity of saturation highlights the importance of reflexivity in qualitative research. Reflexivity is the awareness and critical examination of biases and assumptions by the researcher throughout the study, which helps the researcher have a better understanding of their perspectives and their impact on data interpretation (Charmaz, 2006; Strauss & Corbin, 2014).

#### 3.6.8 Need for detailed systematic analysis

Achieving data saturation requires systematic analysis, coding, and precise identification of themes and patterns in the data. This process requires multiple cycles of coding and analysis, ongoing discussions with other researchers, and the use of various data collection methods such as interviews, observations, document analysis, field notes, and memos to fully grasp the complexity of the phenomenon under study and confidently present all aspects of the phenomenon in the data (Bowen, 2008; Charmaz, 2006; Fusch & Ness, 2015). To achieve this, researchers not only use purposeful and theoretical sampling methods to find information-rich samples depending on the type of study but also employ precise data analysis methods such as comparative analysis and negative case analysis. The use of negative case analysis, which involves questioning or contradicting emerging theories with sources of data, can enhance the credibility and accuracy of the study and assist the researcher in ensuring the validity of their analysis in theory development (Saunders et al., 2018).

### 3.7 Data collection for identifying the antecedents and consequences of the phenomenon

#### 3.7.1 Antecedents

Antecedents are events that occur before the occurrence of the phenomenon under study and have an impact on its occurrence. The question used in this phase is: "Which events or phenomena have been associated with the concept in the past?” (Rogers, 2000). Antecedents of data saturation in this study appeared in two categories, namely study-related factors and researcher-related factors. Table 3 shows these antecedents with references used to support them (See Table 3).

**Table 3:** Antecedents of the concept of saturation.

#### 3.7.2 Study-related factors

To achieve saturation or determine the appropriate sample size for their study, researchers need to be familiar with factors that influence saturation. The reviewed studies showed that study characteristics, including the research objective and question, nature and sensitivity of the phenomenon, characteristics of the research population, sample size, data collection and analysis methods, and available time and resources, all influence the attainment of saturation.

#### 3.7.3 Research purpose or question

The research objective and question have an impact on the time required to achieve saturation in qualitative research. A clear and focused objective or question can expedite the attainment of saturation, while a broad objective or question may require more time for data collection and reaching saturation. (Aldiabat & Le Navenec, 2018; Francis et al., 2010; Fusch & Ness, 2015; Guest et al., 2006; Hennink et al., 2017; Mwita, 2022; Thomson, 2010) Sometimes, the research question may be so extensive that even with continuous data collection, the researcher keeps discovering new features and dimensions of the phenomenon under study, making it difficult to achieve data saturation. Strauss and Corbin suggest that in such cases, instead of prematurely ending data collection and claiming saturation, the researcher should acknowledge this as a limitation of the study (Strauss & Corbin, 2014). They propose that reading several initial interviews and analyzing the data simultaneously can help the researcher understand the essence of the phenomenon and narrow down the focus of the study (Strauss & Corbin, 2014).

#### 3.7.4 Nature and sensitivity of the phenomenon under study

Another aspect that affects the achievement of saturation is the nature and sensitivity of the phenomenon under study (Aldiabat & Le Navenec, 2018; Fusch & Ness, 2015; Hennink & Kaiser, 2022; Morse, 2015b; Thomson, 2010). The novelty, complexity, or abstract nature of the phenomenon requires the researcher to collect extensive data using different methods and spend more time collecting data, meaning that data saturation may be achieved slowly (Charmaz, 2006; Francis et al., 2010; Fusch & Ness, 2015; Guest et al., 2020; Hennink et al., 2017; Morse, 2015b). Additionally, in cases where the phenomenon under investigation is sensitive, such as cases that involve personal values and beliefs, participants may be unwilling to involve others in them, and the researcher may need to conduct more interviews with each participant to create an open and trustworthy environment for obtaining richer information from the participant (Hennink et al., 2017; Thomson, 2010).

#### 3.7.5 Characteristics of the research population

The characteristics of the research population and sample selection play a crucial role in achieving saturation in qualitative research. In comparison to a homogeneous population, a diverse sample in the study population requires the collection of more data to encompass all perspectives and experiences, thus increasing the likelihood of reaching saturation (Guest et al., 2006; Hennink et al., 2017; Hennink et al., 2019; Morse, 2015b; Mwita, 2022). Additionally, selecting samples that provide richer information about the phenomenon under study facilitates access to saturation. This is why participant selection, unlike quantitative studies that are conducted randomly, is done using non-probability sampling techniques such as purposive sampling (selecting a sample that has the most information about the phenomenon under study) and theoretical sampling (samples that contribute to theory formation, completing categories, and their relationships) in qualitative research (Bowen, 2008; Francis et al., 2010; Hennink & Kaiser, 2022; Hennink et al., 2017; Mwita, 2022).

#### 3.7.6 Sample size

The sample size is always a topic of discussion among qualitative researchers (Guest et al., 2020; Hennink & Kaiser, 2022; Hennink et al., 2017; Hennink et al., 2019; Mwita, 2022). Since qualitative research often uses a small sample size, ensuring the adequacy of the sample size to achieve saturation is important. Although there is no fixed rule for sample size in qualitative research, some researchers suggest using a sample size of 12-15 participants, especially in relatively homogeneous populations, to achieve saturation (Guest et al., 2006; Hennink & Kaiser, 2022). Some researchers believe that a larger sample size helps with identifying patterns and themes more effectively by increasing the diversity of perspectives and experiences presented in the data (Morse, 2015b), and increasing the sample size increases the chances of reaching data saturation (Mwita, 2022). However, larger qualitative samples that exceed the necessary limit come with ethical issues such as wasting research funds, burdening participants excessively, and wasting time. On the other hand, sample sizes that are too small to reach saturation also reduce the credibility of study findings (Hennink & Kaiser, 2022). Therefore, researchers recommend reducing the sample size by carefully selecting information-rich samples and striving to obtain rich and diverse information from the samples (Hennink & Kaiser, 2022; Hennink et al., 2017; Marshall et al., 2013; Mwita, 2022), through multiple interviews with one participant (Thomson, 2010) or using unstructured interviews (Morse, 2015b).

#### 3.7.7 Data collection and analysis methods

The methods used for data collection and analysis can significantly impact reaching saturation. Researchers believe that using more than one data collection tool increases the chances of reaching saturation. The use of combined data collection methods such as interviews, focused group discussions, observations, field notes, etc., helps the researcher provide a rich description of the how and why of the phenomenon from the data and reach saturation faster than when using a single method for data collection (Creswell & Poth, 2016; Fusch & Ness, 2015; Guest et al., 2006; Hennink & Kaiser, 2022; Hennink et al., 2017; Marshall et al., 2013; Mwita, 2022). This method is called triangulation (using multiple methods) and is not only limited to data collection but also has applications in sample selection, data analysis, and even controlling for credibility (Bowen, 2008; Hennink et al., 2017).

#### 3.7.8 Time and resources availability

Resources such as time and money can also be influential factors in saturation in qualitative research. Qualitative research is not only time-consuming but also expensive, as the sample size, length of sessions, and use of different data collection methods are influenced by the researcher's available time, financial resources, and material resources (Mwita, 2022). For example, the more time a researcher spends on data collection in each session, the more diverse and varied data they can gather, increasing the chances of reaching saturation (Morse, 2015b; Mwita, 2022). On the other hand, lengthy interviews can lead to participant fatigue or decreased efficiency due to repetitive data collection (Mason, 2010). Therefore, researchers should strive to strike a balance between collecting diverse and comprehensive data while avoiding information overload (Mwita, 2022). Additionally, conducting a qualitative study that has reached saturation requires researchers, especially novice researchers, to have extensive preparation and training, including theoretical knowledge, interviewing skills, and qualitative research techniques, which may require investment in resources such as books, online resources, workshops, and training sessions (Aldiabat & Le Navenec, 2018; Bowen, 2008).

### 3.8 Researcher-related factors

#### 3.8.1 Researcher skill, knowledge and experiences, and Researcher bias

The skills, experience, and knowledge of the researcher are also factors that can affect the number of required samples and the ability to reach saturation. A skilled researcher, using rich interviews, requires a smaller sample size because they can ask effective questions that guide the research and obtain rich information by omitting unnecessary details, guiding the participant, and encouraging them to provide relevant information (Aldiabat & Le Navenec, 2018; Guest et al., 2006; Guest et al., 2020; Thomson, 2010). The researcher's personal experiences and background can also influence their interpretation of the data and their ability to reach saturation. For example, a researcher's cultural background may impact their understanding of specific concepts or topics (Charmaz, 2006). Furthermore, the researcher's beliefs or biases about the studied phenomenon may lead to overlooking important information in data analysis or premature conclusions about saturation by preventing the recognition of emerging themes (Bowen, 2008; Creswell & Poth, 2016; Marshall et al., 2013). Therefore, before starting qualitative research, researchers must ensure that they have sufficient skills and knowledge in interviewing participants, interpreting data, and recognizing what exists in the data (Morse, 2015b). They should also strive to be aware of their biases, perspectives, and worldview and set aside their personal lens when interpreting the data (Fusch & Ness, 2015).

#### 3.8.2 Consequences

Rogers (2000) defines consequences as events that occur as a result of concept. In this phase of analysis the researcher answers this question: What happens after or as a result of the concept? (Rogers, 2000). Literature review shows that saturation affects the quality, credibility, generalizability, transferability, and accuracy of qualitative studies. In addition, saturation leads to saving or wasting time, energy, and costs for researchers.

#### 3.8.3 Ensuring credibility and quality in qualitative research

Reaching partial saturation is vital in qualitative research as it helps ensure the strength and validity of data collection (Bowen, 2008; Fusch & Ness, 2015; O'reilly & Parker, 2013). Morse (2015) considers saturation as a key to quality research and a guarantee of credibility and accuracy in qualitative studies (Morse, 2015b). Saturation is important because it shows that a sample is sufficient for the studied phenomenon and all aspects of the phenomenon under study have been investigated and the collected data effectively demonstrate the diversity, depth, and subtle differences of the subject under study, thereby confirming the content validity (Francis et al., 2010). On the other hand, the inability to achieve data saturation negatively affects the credibility of a study's results (Fusch & Ness, 2015; Kerr et al., 2010). Therefore, studies recommend that researchers facilitate reaching saturation by employing various strategies such as prolonged engagement, continuous observation and thick description, inter-rater reliability, analysis of negative cases, peer debriefing or review, researcher reflexivity, member checking, external audits, and triangulation (Bowen, 2008; Fusch & Ness, 2015; Hennink et al., 2017; Morse, 2015b).

#### 3.8.4 Saving time, energy, and budget

Another consequence of reaching saturation is timely estimation and stopping data collection, which prevents wasting time, resources, and energy. Conversely, failure to timely recognize saturation and introducing qualitative samples with higher volumes wastes the budget for qualitative research and leads to accumulating additional and heavier information, resulting in wasted time and energy for researchers in analyzing the data. Premature saturation also fails to fully identify phenomena with very small sample sizes and leads to a decrease in research credibility and wasted resources (Hennink et al., 2017; Mwita, 2022).

**Fig. 2:**
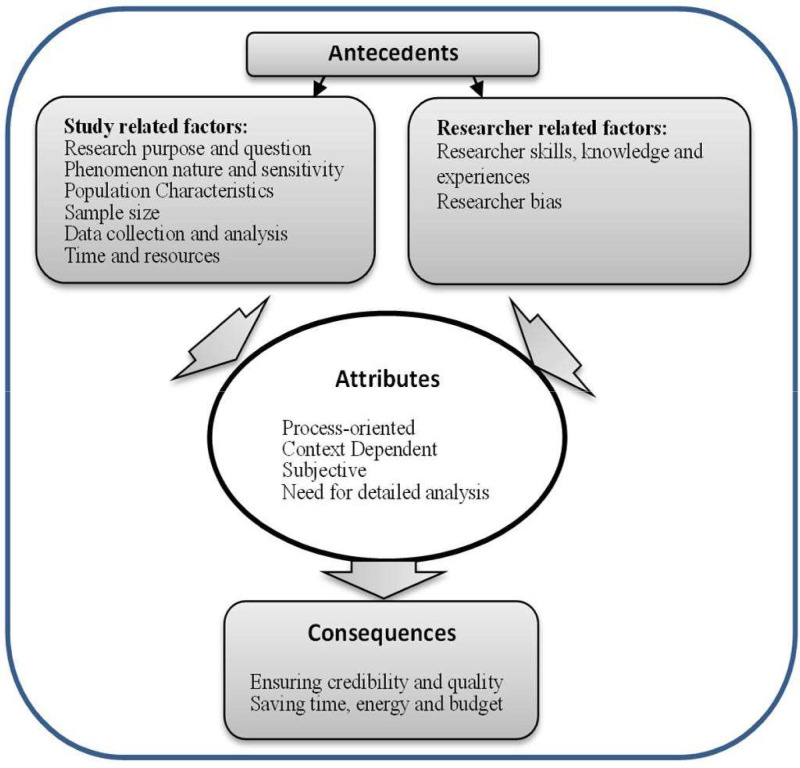
Conceptual model of concept analysis of saturation in qualitative research.

A conceptual model is presented in Fig. 2, based on study findings, to better understand the concept of saturation (See Fig. 2).

#### 3.8.5 Identification of an exemplar if appropriate

In order to provide a thorough understanding of the concept, examples are often utilized. These examples are drawn from real-world data and are deemed valuable if they aid in the analysis (Tofthagen & Fagerstrøm, 2010). However, due to the abstract nature of saturation, it was not feasible to provide a practical exemplar.

#### 3.8.6 Strengths and limitations

This study contributes to expanding the knowledge base on the concept of saturation by thoroughly examining and elucidating this concept. Furthermore, discussing the influence of research-related factors on saturation enables researchers to attain genuine saturation by managing these factors. The use of concept analysis to elucidate the concept of saturation is also a noteworthy aspect of this study. Using this approach not only enhances the credibility of research method but also presents a systematic application of this method for analyzing a concept. A limitation of this study was that we only included studies that were published in English, which may exclude other published studies on saturation. Likewise, the exclusion of unpublished studies or gray literature may lead to the exclusion of other insights into the concept of saturation. In order to overcome these limitations, the researchers attempted to assess a broader range of studies, using broad keywords and separate searches performed by two investigators.

## 4 Conclusion

Saturation is a vital concept in qualitative research that allows a comprehensive understanding of the phenomenon under study. In this study, using Rogers' evolutionary concept analysis, we investigate the concept of saturation and discuss its types, attributes, antecedents, and its consequences. A qualitative researcher needs to articulate the saturation and its type and scope in their research, and with a clear understanding of the factors that affect saturation, contribute to reducing the sample size and saving on budget and study time. The results of this study showed that compliance with some factors facilitates reaching saturation. These factors include: selecting a clear and focused research objective or question, paying attention to the nature and sensitivity of the phenomenon under study in data collection, selecting information-rich samples at the beginning of data collection, using triangulation in data collection and data analysis. It is recommended that qualitative researchers explain saturation, meaning and how to achieve to deal with the criticism of qualitative studies and to guarantee the validity of their study. Future studies are needed to investigate how the antecedents affect reaching saturation and sample size estimation.

## Ethical Approval

Ethical approval was obtained from Qazvin University of Medical Sciences Ethics Committee (IR.QUMS.REC.1402.180).

## Funding source

This research received financial support from Vice Chancellor for Research and Technology of Qazvin University of Medical Sciences.

## CRediT authorship contribution statement

**Sara Rahimi:** Writing – review & editing, Validation, Supervision, Project administration, Methodology, Investigation, Formal analysis, Conceptualization. **Marzieh khatooni:** Writing – original draft, Methodology, Investigation, Conceptualization.

## Declaration of competing interest

None.
